# Decision-making factors and their thresholds for total knee arthroplasty in lateral tibiofemoral osteoarthritis patients: a retrospective cohort study

**DOI:** 10.1186/s43019-022-00168-w

**Published:** 2022-10-23

**Authors:** Byung Sun Choi, Jung Min Kim, Hyuk-Soo Han

**Affiliations:** 1 Department of Orthopaedic Surgery, Seoul National University Hospital, 101 Daehak-ro, Jongno-gu, Seoul, 03080 Republic of Korea; 2 Department of Orthopaedic Surgery, Seoul National University College of Medicine, Seoul, Republic of Korea

**Keywords:** Lateral tibiofemoral arthritis, Decision-making factor, Tibiofemoral subluxation, Total knee arthroplasty

## Abstract

**Background:**

There has been no study examining lateral tibiofemoral (TF) osteoarthritis (OA) and objective decision-making factors affecting when patients decide to have total knee arthroplasty (TKA). The purpose of this study was to assess which factors and their thresholds cause patients with lateral TF OA to decide on TKA.

**Methods:**

We conducted a retrospective cohort study and identified patients who had initially been diagnosed with isolated lateral TF OA from October 2004 to February 2021. We finally included 56 patients; patients who had chosen conservative treatment followed by in-depth interviews for the deliberation stage (*n* = 32), and the other patients who decided to undergo TKA for the decision-making stage (*n* = 24). Demographic, clinical, and radiographic characteristics were considered candidate predictive factors. Radiographic variables included the Ahlbäck grade, hip–knee–ankle (HKA) angle, joint line convergence angle (JLCA), and TF subluxation. Univariate and multivariate logistic regression analyses were performed.

**Results:**

Clinically, the pain visual analog scale (VAS) score was significantly higher and the knee flexion angle was lower at the decision-making stage. Radiographic measurements showed that the Ahlbäck grade, HKA angle, JLCA, and TF subluxation measured at the center, in addition to the tibiotalar angle, differed statistically between the two stages. According to univariate analyses, two clinical characteristics and six radiographic variables on the ipsilateral side of the leg, and one radiographic variable on the contralateral side of the leg were included as factors influencing the patients’ decision to undergo TKA. After making adjustments based on multivariate analysis, the ipsilateral knee pain VAS (OR = 1.61; 95% CI = 1.14–2.28, *p* = 0.007) and medial TF subluxation measured at the center (OR = 1.14, 95% CI = 1.01–1.32, *p* = 0.072) were found to be significant factors for choosing TKA. The area under the curve (AUC) for pain VAS was 0.757 and the cutoff value was 4.5. The AUC for TF subluxation measured at the center was 0.697 and the cutoff value was −4.10% of medial TF subluxation.

**Conclusion:**

Higher ipsilateral knee pain VAS and more severe medial TF subluxation measured at the center were independent factors affecting patient decisions to undergo TKA with lateral TF OA. Understanding the determining factors that may affect patient decision-making when considering TKA may be an essential aspect of evaluating the prognosis of patients with lateral TF OA.

**Level of evidence:**

III.

**Supplementary Information:**

The online version contains supplementary material available at 10.1186/s43019-022-00168-w.

## Introduction

Knee osteoarthritis (OA) patients who have failed conservative treatment are currently treated with osteotomy, unicompartmental knee arthroplasty (UKA), or total knee arthroplasty (TKA) [1]. However, there are few definite criteria for when an OA patient should have knee surgery and which surgery to receive. A surgeon can explain the benefits of knee surgery to patients and help them determine whether knee surgery is necessary. So far, we have little information about the factors and thresholds that affect patient decisions to undergo total knee arthroplasty.

Previous studies examining how patients make decisions about TKA have mostly been qualitative studies using subjective factors such as pain and patient expectations [2]. Until the patient reaches the decision-making threshold [3], they are considered to be in the deliberation process. Reaching this threshold defines the decision-making process [4]. However, previous studies did not distinguish between medial or lateral tibiofemoral (TF) OA, and there has been no analysis of objective decision-making factors [5].

When considering the three compartments of the knee joint, isolated lateral compartment knee OA is relatively less common and causes fewer clinical problems [6, 7]. Most activities of daily living generate a greater load in the medial compartment than the lateral compartment of the knee in neutral or varus alignment [8]. This difference in lead has been used to explain the finding that medial knee OA is more prevalent overall than lateral knee OA [9]. Most studies have focused on medial knee OA, and to our knowledge, few studies have examined the factors determining surgery.

There have been previous studies evaluating the relationship between TF OA severity and radiographic morphology or alignment [9–12]. To our knowledge, there has been no study of lateral TF OA and objective decision-making factors about when patients decide to undergo TKA. Finding predictive factors for patients with lateral TF OA to make decisions to undergo surgery is important because these factors are potentially modifiable and used for prevention and consultation. The hypothesis of this study is that there would be some different factors between patients with conservative treatment and patients with the decision of surgery. The purpose of this study was to assess which factors and their thresholds cause lateral TF OA patients to decide to undergo TKA by reviewing demographic, clinical, and radiographic characteristics.

## Materials and methods

### Study design and patients

**Fig. 1:**
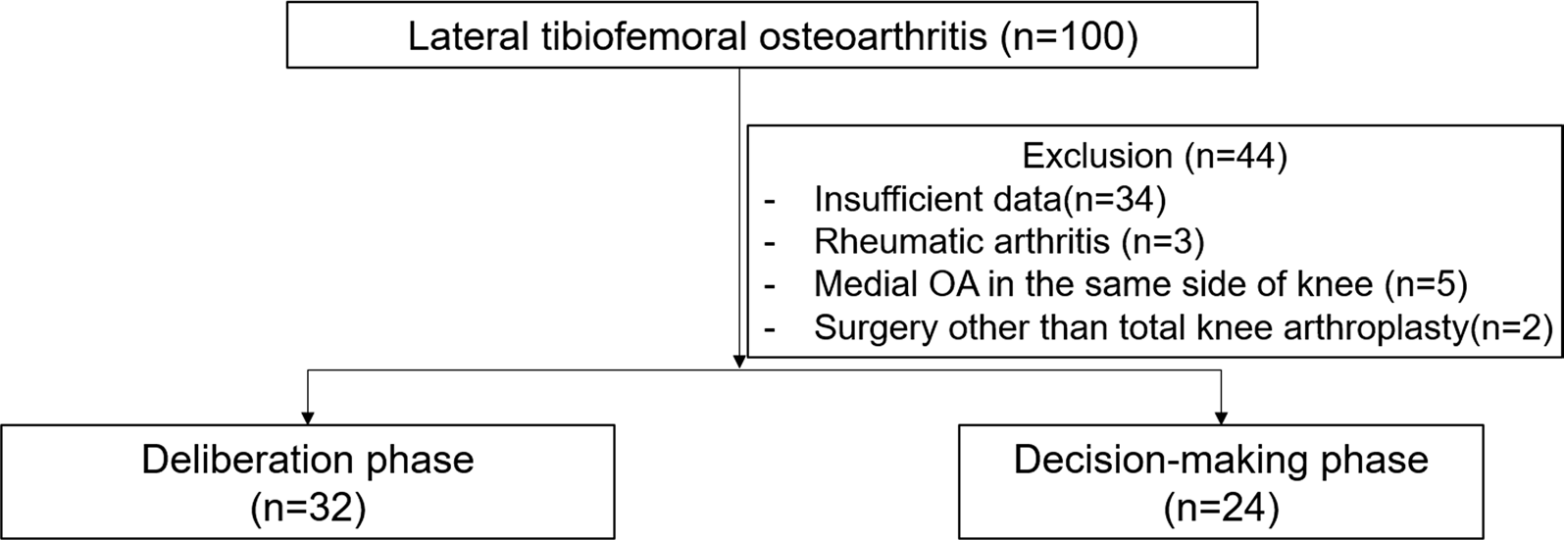
Study diagram illustrating the selection of study participants in the retrospective cohort design

This retrospective cohort study was approved by the institutional review board (IRB number: 2104-232-1217). We identified 100 patients who had initially been diagnosed with lateral TF OA by one surgeon at a tertiary referral hospital from October 2004 to February 2021. Of those patients, 44 patients were excluded due to insufficient data, history of rheumatoid arthritis, combined medial TF OA on the same side of the knee, or history of non-TKA surgery (Fig. 1). All patients had a previous history of conservative treatment at least 6 months involving patient education for weight control and exercise, antiinflammatory and analgesic medications, or intraarticular injection. Information about knee surgeries was presented to the patients along with nonsurgical options, alternative therapies, and the risks and benefits of surgery. The patients carefully chose between surgical or nonsurgical treatments after an in-depth interview. Finally, 56 patients were included, and these were classified into two groups: one group decided to follow-up with conservative treatment on an outpatient basis as the deliberation stage, and another group decided to undergo surgery as the decision-making stage. Surgeries for lateral TF OA included TKA (Fig. 1). In the deliberation stage, all variables were collected and analyzed at the last outpatient follow-up and in the decision-making stage, all variables were analyzed at the outpatient clinic when surgery was decided.

### Demographics and clinical characteristics

For the clinical characteristics of lateral TF OA patients, we collected the following variables from the medical records: sex, age, body mass index (BMI), comorbidity, OA grade of contralateral knee, duration of symptoms, pain visual analog scale (VAS), history of trauma, previous steroid injection at the knee joint or partial meniscectomy, and range of knee motion.

### Radiologic measurement

All radiographic variables were measured by two authors. The intra- and interrater reliabilities were tested, and the remeasurements were done after 1 month from the first measurement. All radiographic images were acquired using a picture archiving and communication system (PACS) (Maroview 5.4, Infinitt, Seoul, South Korea), and assessments were made with the PACS software.

To separately evaluate the grade of arthritic severity and bone attrition in each of the three compartments, we adopted the modified Ahlbäck grading system [13]. The medial and lateral compartment scores were defined as the presence of joint space narrowing (1 point) or obliteration (2 points), tibial and/or femoral sclerosis (0.5 points each), osteophytes < 1 cm (0.5 points) or > 1 cm (1 point), or joint subluxation (1 point). The patellofemoral compartment scores were defined as the presence of joint space narrowing (1 point) or obliteration (2 points), osteophytes < 1 cm (0.5 points) or > 1 cm (1 point), translation of the patella (1 point), or attrition (1 point). Further, we assigned the highest scores of the three compartments. Knees with a score of ≤ 2 points in all compartments were classified as mild arthritis. Knees with a score of > 2 and < 4 points in at least one compartment were graded as moderate arthritis. Knees with a score of ≥ 4 points in at least one compartment were graded as severe arthritis.

**Fig. 2:**
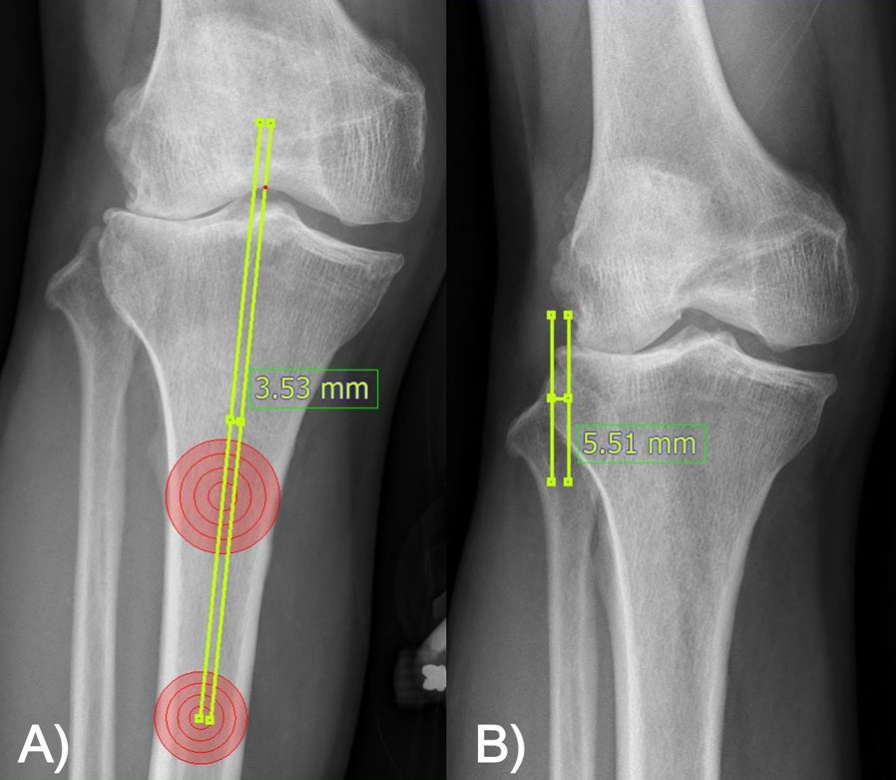
**A** Tibiofemoral subluxation in the lateral cortex in Rosenberg view was measured. **B** Tibiofemoral subluxation in the center from the standing anteroposterior view was measured. When the tibia subluxed to the medial direction, it was defined as a positive value

To determine the knee joint radiographic parameters related to the lateral TF OA, we measured the hip–knee–ankle (HKA) angle from full-length standing views [14], the joint line convergence angle (JLCA), the TF subluxation measured in the lateral cortex [15], and the TF subluxation measured at the center (Fig. 2) [16]. TF subluxation was measured using the both knee anteroposterior (AP) standing and the Rosenberg view. The Rosenberg view was obtained using a posteroanterior (PA) radiograph with weight-bearing and 45° of knee flexion. TF subluxation was measured in the lateral cortex by dividing the distance between the line of the most lateral border of the femoral condyle and the tibial lateral condyle by the tibial plateau length. If the tibia subluxed more medially, it was measured as a positive value for medial TF subluxation. TF subluxation measured at the center was defined as the distance between the line parallel to the tibial anatomical axis and the apex of the intercondylar notch of the femur divided by the tibial plateau length. If the tibial anatomical axis subluxed more medially, it was measured as a positive value. TF subluxation measured in the lateral cortex and the center were divided by the tibial plateau length, and the values were obtained as percentages.

**Fig. 3:**
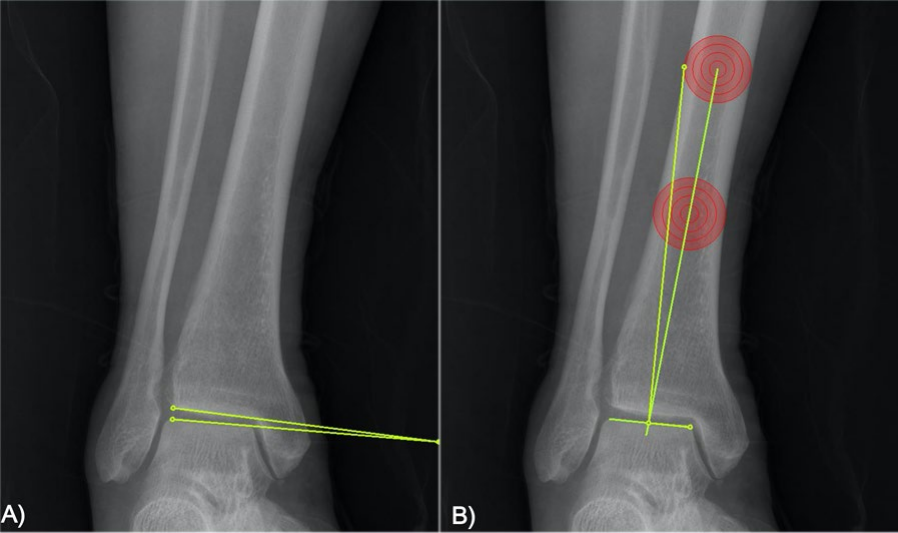
The measurement of alignment of the right ankle joint is shown. **A** Tilt angle of the ankle joint and **B** the tibiotalar angle were measured. The ankle was in varus alignment, it was assessed as a positive value

We measured pelvic geometry and ankle alignment [17] for all patients (Fig. 3). Head–head length (HHL) of the hip joint was defined as the distance between the two femoral centers. Femoral offset (FO) was defined as the distance between the line parallel to the femoral diaphyseal axis and the center of the femoral head. Pelvic obliquity was measured by the Osebold technique [18]. Line 1 was drawn between the superior aspects of the iliac crest while line 2 was drawn parallel to the plane. When the side of interest of the iliac crest rose upward, it was measured as a positive value. The neck shaft angle (NSA) of the femur was defined as the angle between the femoral diaphyseal axis and the femoral head-to-neck axis. To define ankle alignment, the tilt angle of the ankle (TAA) was measured as the angle between the horizontal line and the talar dome. The tibiotalar angle (TTA) was measured as the angle between the tibial diaphyseal axis and the line drawn to the talar dome. If the ankle had varus alignment, TAA and TTA were assessed as positive values. We also measured the Kellgren–Lawrence (K–L) grade of the hip and ankle joints.

### Statistical analysis

Continuous data were reported in terms of the mean and standard deviation, and analyzed using the Student’s *t*-test or Mann–Whitney *U* test after the Kolmogorov–Smirnov normality test. Categorical data were reported as the number and percentage of patients, and it was analyzed using the Fisher exact test or the chi-squared test.

Univariate logistic regression analyses were conducted to evaluate the effect of the variables. Multicollinearity was checked for dependent variables using a variance inflation factor. Then, the variables that were found to be significant or nearly significant (*p* < 0.1) in univariate logistic regression analyses were included in a backward-stepwise multivariate logistic regression analysis. We reported an odds ratio (ORs) with a 95% confidence interval (CI) for all associations, and a *p*-value of < 0.05 was considered significant. The sensitivity and specificity of the receiver operating characteristic (ROC) curve and the area under the curve (AUC) were calculated for significant variables on multivariate logistic regression. The cutoff value was calculated based on sensitivity and specificity. A post-hoc power analysis was performed to calculate the power of the present study (1 − *β* probability). The intra- and interrater reliabilities of the radiographic measurements were assessed using the intraclass correlation coefficient, which can be used to quantify the variability between measurements. In general, values of > 0.75 are considered to be in good agreement, while values of < 0.5 are considered to represent poor agreement [19]. We used SPSS software (Version 26.0; IBM Co., Chicago, IL, USA) for the statistical analysis.

## Results

Of the 24 patients in the decision-making stage, 13 patients (54%) changed their decision to surgery during outpatient follow-up, and 11 patients (46%) decided to have surgery at the first outpatient visit. Table 1 summarizes the patient demographic and clinical characteristics. The ipsilateral side pain VAS was statistically higher (*p* = 0.002) and the further flexion angle was smaller in the decision-making stage (*p* = 0.01). The radiographic parameters between these two stages are described in Table 2. Intrarater reliability (0.92–0.96) and interrater reliability (0.77–0.90) were measured, and they achieved satisfactory results. In the decision-making stage, the Ahlbäck OA grade was higher, and the valgus HKA angle and valgus JLCA were observed. This refers to the medial joint opening. Patients who opted for surgery showed less lateral translation, but the results measured at the lateral cortex showed more lateral subluxation. They also showed a greater varus TTA. Since the radiographic parameters of the contralateral leg could affect the ipsilateral side of lateral TF OA, they are assessed in Additional file 1: Supplement table 1. There was no statistical difference between the two stages.

**Table 1:** Demographic and clinical characteristics of the patients with lateral tibiofemoral osteoarthritis

	Deliberation stage (*n* = 32)	Decision-making stage (*n* = 24)	*p*-Value
Sex^*^			
Male	11 (34%)	7 (29%)	n.s.^†^
Female	21(66%)	17 (71%)	
Age (years)^‡^	66.53 ± 11.76	69.33 ± 6.22	n.s.^§^
BMI (kg/m^2^)^‡^	24.82 ± 2.41	25.82 ± 1.93	n.s.^∥^
Diabetes mellitus^*^	2 (6%)	4 (17%)	n.s.^¶^
Depression disorder^*^	0 (0%)	2 (8%)	n.s.^¶^
Angina	0 (0%)	0 (0%)	n.s
Cerebrovascular accident	4 (12.5%)	0 (0%)	n.s.^¶^
Site of osteoarthritis (year)^*^			
Right	19 (59%)	14 (58%)	n.s.^†^
Left	13 (41%)	10 (42%)	
Bilaterality of lateral knee OA	6 (18.8%)	8 (33%)	n.s.^†^
OA site of contralateral side of knee			
None	26 (81.3%)	13 (54%)	n.s.^†^
Medial OA	0 (0%)	3 (11%)	
Lateral OA	6 (18.8%)	8 (31%)	
Duration of symptoms (months)	46.94 ± 58.71	47.80 ± 75.86	n.s.^§^
Follow-up period (years)	3.73 ± 2.13	3.60 ± 2.55	n.s.^§^
Ipsilateral side pain VAS^‡^	4.06 ± 1.87	5.79 ± 2.06	0.002^§,**^
Contralateral side pain VAS^‡^	3.94 ± 1.32	4.33 ± 2.70	n.s.^§^
Trauma history^*^	11 (34%)	4 (17%)	n.s.^†^
Injection history^*^	20 (63%)	20 (83%)	n.s.^†^
Meniscectomy history^*^			
None	20 (63%)	20 (83%)	n.s.^†^
Ipsilateral	8 (25%)	3 (13%)	
Contralateral	4 (12%)	1 (4%)	
Range of knee motion (°)^‡^			
Flexion contracture	2.14 ± 3.93	5.24 ± 8.29	n.s.^§^
Further flexion	135.00 ± 4.08	123.10 ± 15.29	0.01^§^^,^^**^

*The values are given as the number of patients

^†^Pearson’s chi-square test

^‡^Values are given as the mean and standard deviation

^§^Mann–Whitney *U* test

^∥^Independent *t*-test

^¶^Fisher’s exact test

^**^Statistically significant

*BMI* body mass index, *OA* osteoarthritis, *VAS* visual analog scale

**Table 2:** Comparison of radiologic parameters between deliberation phase and decision-making phase

	Deliberation stage (*n* = 32)	Decision-making stage (*n* = 24)	*p*-Value
Pelvis			
LLD (mm)^*^	−1.13 ± 6.67	−2.26 ± 8.64	n.s.^†^
Pelvic obliquity (°)^*^	−0.30 ± 1.97	−0.66 ± 2.57	n.s.^†^
Knee joint			
Ahlbäck grade^‡^			
Mild	7 (22%)	3 (12%)	0.003^§,^^∥^
Moderate	18 (56%)	5 (21%)	
Severe	7 (22%)	16 (67%)	
HKA (°)^*^	− 1.53 ± 5.47	− 8.46 ± 7.74	< 0.001^¶,^^∥^
JLCA (°)^*^	− 0.44 ± 2.80	− 2.70 ± 4.19	0.028^¶,^^∥^
TF subluxation at the lateral cortex (%)^*^			
Knee AP	− 2.46 ± 2.7	− 3.53 ± 4.66	n.s.^†^
Rosenberg view	− 0.86 ± 3.38	− 3.82 ± 5.86	0.033^¶,^^∥^
TF subluxation at the center (%)^*^			
Knee AP	− 5.63 ± 5.63	− 0.61 ± 7.46	0.006^†,^^∥^
Rosenberg view	− 1.02 ± 4.64	−1.51 ± 9.12	n.s.^†^
Hip joint			
K–L grade of hip^‡^			
Mild	27 (84%)	23 (96%)	n.s.^§^
Severe	5 (16%)	1 (4%)	
HHL (mm)^*^	181.10 ± 7.82	185.85 ± 11.55	n.s.^¶^
FO (°)^*^	42.60 ± 5.39	41.09 ± 5.14	n.s.^†^
NSA (°)^*^	54.36 ± 5.10	53.49 ± 5.70	n.s.^†^
Ankle joint			
K–L grade of ankle^‡^			
Mild	31 (97%)	23 (96%)	n.s.^**^
Severe	1 (3%)	1 (4%)	
TAA (°)^*^	− 0.37 ± 1.49	− 0.05 ± 1.56	n.s.^†,^^∥^
TTA (°)^*^	0.58 ± 3.81	3.71 ± 5.47	0.021^¶,^^∥^

*The values are given as the mean and standard deviation

^†^Independent *t*-test

^‡^The values are given as the number of patients

^§^Pearson’s chi-square test

^∥^Statistically significant

^¶^Mann–Whitney *U* test

^**^Fisher’s exact test

*LLD* leg length discrepancy, *HKA* hip–knee–ankle angle, *JLCA* joint line convergence angle, *AP* anteroposterior, *K–L* Kellgren–Lawrence, *HHL* head–head length of pelvis, *FO* femoral offset, *NSA* neck shaft angle of hip, *TAA* tilt angle of ankle, *TTA* tibiotalar angle

In univariate logistic regression analyses, two clinical variables and seven radiographic variables were found to be the factors affecting the patients’ decision to receive surgery (Table 3). Before conducting multivariate logistic regression analysis, multicollinearity was checked, and no interaction or multicollinearity effects were found (Additional file 2: Supplement table 2). The results of the multivariate logistic regression analysis are presented in Table 4. After adjusting for other factors, ipsilateral leg pain VAS (OR = 1.61; 95% CI = 1.14–2.28, *p* = 0.007) and more medial TF subluxation measured at the center (OR = 1.14; 95% CI = 1.01–1.27, *p* = 0.031) were significant decision-making factors for receiving surgery. The post-hoc power analysis calculated on the sample size was found to be 78.9% using a two-sample and two-sided *t*-test (number, mean, and standard deviation of TF subluxation in the center for the deliberation stage and decision-making stage were 32, −5.63 ± 5.63, 24, and −0.61 ± 7.46, respectively).

**Table 3:** Independent risk factors of decision for surgery in lateral tibiofemoral osteoarthritis according to the univariate logistic regression analysis

Variables	Crude ORs (95% CI)	*p*-Value
Sex	1.27 (0.41–3.99)	0.680
Age	1.03 (0.97–1.09)	0.290
BMI (kg/m^2^)	1.24 (0.96–1.60)	0.104
Diabetes mellitus	3.00 (0.50–17.95)	0.229
Depression disorder	1.00 (1.00–1.00)	0.999
Angina	1.00 (1.00–1.00)	0.999
Cerebrovascular accident	1.00 (1.00–1.00)	0.999
Site of osteoarthritis (right versus left)	1.04 (0.36–3.06)	0.938
Bilaterality of lateral knee OA	2.17 (0.63–7.40)	0.217
OA site of contralateral side of knee (none versus lateral OA)	2.67 (0.76–9.31)	0.124
Duration of symptoms (months)	1.00 (0.99–1.01)	0.961
Follow-up period	0.98 (0.77–1.23)	0.830
Trauma history	0.38 (0.10–1.40)	0.146
Injection history	3.00 (0.83–10.90)	0.095
Meniscectomy history (none versus ipsilateral)	0.38 (0.09–1.62)	0.189
Range of knee motion		
Flexion contracture	1.08 (0.91–1.29)	0.358
Further flexion	0.83 (0.69–1.01)	0.104
LLD	0.98 (0.91–1.05)	0.577
Pelvic obliquity	0.93 (0.73–1.18)	0.550
Ipsilateral side of lower extremity		
Pain VAS	1.59 (1.15–2.18)	0.005^*^
Ahlbäck grade (mild versus severe)	5.33 (1.06–26.90)	0.043^*^
HKA	0.85 (0.77–0.94)	0.002^*^
JLCA	0.83 (0.71–0.98)	0.025^*^
TF subluxation at the lateral cortex		
Knee AP	0.93 (0.80–1.07)	0.302
Rosenberg view	0.86 (0.75–0.99)	0.032^*^
TF subluxation at the center		
Knee AP	1.13 (1.03–1.25)	0.012^*^
Rosenberg view	0.99 (0.92–1.07)	0.791
K–L grade of hip joint (mild versus severe)	0.24 (0.03–2.16)	0.200
HHL	1.06 (0.99–1.12)	0.120
FO	0.95 (0.85–1.05)	0.288
NSA	0.97 (0.88–1.07)	0.542
K–L grade of ankle joint (mild versus severe)	1.35 (0.08–22.70)	0.836
TAA	1.15 (0.81–1.65)	0.435
TTA	1.16 (1.02–1.31)	0.020^*^
Contralateral side of lower extremity		
Pain VAS	1.11 (0.85–1.45)	0.465
Ahlbäck grade (mild versus severe)	4.19 (1.10–15.90)	0.035^*^
HKA	1.03 (0.95–1.12)	0.463
JLCA	1.09 (0.93–1.27)	0.289
TF subluxation at the lateral cortex		
Knee AP	0.88 (0.71–1.08)	0.205
Rosenberg view	0.94 (0.80–1.11)	0.464
TF subluxation at the center		
Knee AP	1.06 (0.97–1.17)	0.208
Rosenberg view	1.01 (0.92–1.10)	0.900
K–L grade of hip joint (mild versus severe)	1.00 (1.00–1.00)	0.999
HHL	1.06 (1.00–1.13)	0.104
FO	1.00 (0.91–1.10)	0.989
NSA	1.05 (0.95–1.15)	0.332
K–L grade of ankle joint (mild versus severe)	1.00 (1.00–1.00)	0.999
TAA	0.89 (0.66–1.21)	0.466
TTA	1.06 (0.93–1.20)	0.397

*Statistically significant

*ORs* odds ratio, *CI* confidence interval, *BMI* body mass index, *OA* osteoarthritis, *LLD* leg length discrepancy, *VAS* visual analog scale, *HKA* hip–knee–ankle angle, *JLCA* joint line convergence angle, *AP* anteroposterior, *K–L* Kellgren–Lawrence, *HHL* head–head length of pelvis, *FO* femoral offset, *NSA* neck shaft angle of hip, *TAA* tilt angle of ankle, *TTA* tibiotalar angle

**Table 4:** Multivariate logistic regression analysis by backward stepwise method

Variables	β coefficient	Adjusted ORs (95% CI)	*p*-Value
Injection history	1.274	3.58 (0.36–35.24)	0.275
Ipsilateral side of lower extremity			
Pain VAS	0.479	1.61 (1.14–2.28)	0.007^*^
Ahlbäck grade (mild versus severe)	− 0.516	0.60 (0.03–13.38)	0.745
HKA (°)	−0.097	0.91 (0.65–1.27)	0.568
JLCA (°)	0.062	1.06 (0.74–1.54)	0.744
TF subluxation at the lateral cortex in Rosenberg view (%)	0.022	1.02 (0.78–1.33)	0.872
TF subluxation at the center in knee AP (%)	0.127	1.14 (1.01–1.27)	0.031^*^
TTA (°)	0.133	1.14 (0.99–1.32)	0.072
Contralateral side of lower extremity			
Ahlbäck grade (mild versus severe)	0.996	2.71 (0.28–26.17)	0.390

*Statistically significant

*ORs* odds ratio, *CI* confidence interval, *VAS* visual analog scale, *HKA* hip–knee–ankle angle, *JLCA* joint line convergence angle, *AP* anteroposterior, *TTA* tibiotalar angle

The ROC curve showed that the ipsilateral side of leg pain VAS and TF subluxation measured at the center were useful to determine the cutoff values for the decision-making threshold. The AUC for pain VAS was 0.757 (95% CI = 0.623–0.890, *p* = 0.001), and the cutoff value was 4.5 with a sensitivity of 83.3% and a specificity of 59.4%. The AUC for TF subluxation measured at the center was 0.697 (95% CI = 0.557–0.836, *p* = 0.012), and the cutoff value was − 4.10% of medial TF subluxation with a sensitivity of 70.8% and a specificity of 65.6%.

## Discussion

The most important findings in this study are that the higher ipsilateral leg pain VAS and the more medial subluxation measured at the center are, the more likely it is that lateral TF OA patients would decide to receive TKA. These patients were more likely to decide on TKA if the pain VAS was greater than 4.5 and the medial TF subluxation measured at the center was more than − 4.1%.

In the present study, knee pain VAS was shown to be an independent predictive factor for determining whether to have surgery in adjusted multivariate logistic regression analysis. Previous studies have shown that the main triggers for undergoing TKA are severe pain and inability to walk [5]. Osteoarthritic pain affected their daily life including activities, mood, walking ability, and social activities [20]. When the pain became unbearable, patients were willing to consider knee surgery. However, these findings are from a qualitative analysis that did not distinguish three compartments of knee joint. Our study is meaningful in that we not only ascertained a pain VAS level by limiting it to the lateral TF compartment, but in that we also presented the value of 4.5 as a statistically significant cutoff value for a decision-making threshold for knee surgery.

In a biomechanical model, the contact pressure of the tibiofemoral joint was found to be distributed differently in the OA knee, and this change in contact pressure resulted in load transfer between the cartilage and the meniscus within compartments [21]. The results of an in vivo study showed that OA was correlated with the TF translation change [22]. In addition, TF subluxation, which refers to abnormal displacement of the femur with respect to the tibia, caused impingement of the tibial eminence on the femoral condyle [23]. By contrast, other previous studies have shown that preoperative coronal TF subluxation did not worsen functional outcomes [15] and was not an independent factor for TKA [24]. In the present study, only patients with lateral TF OA were included, and the degree of subluxation was investigated in both the lateral and central aspects of the knee joint. Because tibiofemoral morphological changes varied according to the degree of knee flexion, we measured TF subluxation in the knee full-extension AP and Rosenberg view. Our results showed that lateral subluxation of tibia occurred, but the degree of subluxation measured at the center was small. In the case of lateral TF OA, not only did lateral subluxation occur, but so did three-dimensional rotation around the lateral TF joint as a pivot point. Further, only the central measurement of subluxation should reflect the rotation of the joint and is a meaningful factor in deciding on TKA [22].

Although there have been some studies of valgus knee alignment and lateral TF OA progression, no studies have investigated the factors affecting patient decisions regarding lateral TF OA surgery. In a population-based longitudinal study, the prevalence of lateral TF OA was frequent because of the relatively high prevalence of valgus malalignment [12]. In an animal model, valgus knee alignment may result in knee OA because of loss of cartilage and bone height [25]. In a prospective longitudinal cohort study, valgus knee alignment was found to be associated with an increased risk of lateral TF OA progression [11]. By contrast, in a cadaveric study, lateral joint pressure was not significantly increased by valgus knee alignment [26]. In a prospective study, valgus knee alignment was not associated with incidental lateral damage [27]. In our study, the valgus HKA angle is found to be an independent factor in univariate logistic regression analysis, but not a significant risk factor in multivariate logistic regression analysis. These results suggest that valgus alignment slowly affects the patient’s decision-making process [28], and that factors other than knee alignment may have a greater influence on the surgical decision.

The present study investigated not only the reference knee joint, but also the adjacent hip and ankle joints and the contralateral knee joint. A previous gait analysis showed that knee OA increased ankle varus moment by 50% [29]. Our results showed that TTA, which is indicative of ankle alignment, had more varus features at the decision-making stage. Meanwhile, TAA representing ankle congruency did not differ between the two stages. When a knee joint develops a valgus deformity, the ankle joint appears to compensate for it. However, the patients included in the present study did not show high degrees of joint incongruence or decompensation.

This study has several limitations. First, there is an inherent bias associated with retrospective studies existing. Magnetic resonance (MR) or subjective clinical scores could not be included because they were not measured in all patients. Also, it was not possible to clearly obtain information on partial meniscectomy of lateral meniscus performed in other hospitals and previous trauma history. Therefore, additional studies are needed to elucidate the relationship between OA progression and partial meniscectomy or trauma history in lateral TF OA. In addition, the present study was performed as a cross-sectional study, not a longitudinal cohort. Therefore, OA progression over time was not accounted for. Second, the number of patients was small and sample size calculation was not performed. However, one strength of our study is that lateral TF OA is relatively less frequent than medial TF OA in the Asian population, and this has not been previously investigated. Therefore, the post-hoc power analysis was conducted. Third, patients are predominantly female, and it may be difficult to apply to men. Although women prevailed, there was no difference between the two stages. Fourth, decisions regarding surgery may involve other factors. Previous iterative thematic analysis study has identified nine topics (stress, expectation of outcome, model of care, sources of information, personal situation, mental status, coping strategies, loss of control, and trust in doctor) to aid in patient decision-making regarding TKA [2].

## Conclusion

Higher ipsilateral leg pain VAS and more severe medial TF subluxation measured at the center were found to be independent risk factors for patients with lateral tibiofemoral OA to choose TKA. Understanding all determining factors that may affect the patients’ decision-making process when considering surgery is essential to predicting the isolated TF OA prognosis. This work can provide a basis for understanding lateral TF OA and helping patients and clinicians decide upon a treatment plan.

## Supplementary Information

**Additional file 1.** Supplement table 1.

**Additional file 2.** Supplement table 2.

## Data Availability

The datasets generated and/or analyzed during the current study are available from the corresponding author on reasonable request.

## Abbreviations

TF: Tibiofemoral
OA: Osteoarthritis
VAS: Visual analog scale
OR: Odds ratio
AUC: Area under the curve
UKA: Unicompartmental knee arthroplasty
TKA: Total knee arthroplasty
DFO: Distal femoral osteotomy
BMI: Body mass index
PACS: Picture archiving communication system
HKA: Hip–knee–ankle
JLCA: Joint line convergence angle
AP: Anteroposterior
HHL: Head–head length
FO: Femoral offset
NSA: Neck shaft angle
TAA: Tilt angle of the ankle
TTA: Tibiotalar angle
K–L: Kellgren–Lawrence
CI: Confidence interval
ROC: Receiver operating characteristic
LLD: Leg length discrepancy
VIF: Variation inflation factors
