# Supplementary material for: Decision-making factors and their thresholds for total knee arthroplasty in lateral tibiofemoral osteoarthritis patients: a retrospective cohort study
Source: Knee Surg Relat Res. 2022 Oct 23;34:41. doi: 10.1186/s43019-022-00168-w (PMC9590191; doi:10.1186/s43019-022-00168-w)
Supplement: Supplementary file 1 — Additional file 1. Supplement table 1. [file 43019_2022_168_MOESM1_ESM.docx]

| **Appendix A.** Comparison of contralateral side radiologic parameters between deliberation phase and decision-making phase | | | |
| --- | --- | --- | --- |
|  | Deliberation stage  (n=32) | Decision-making stage  (n=24) | P-value |
| Knee joint |  |  |  |
| Ahlbäck grade^*^ |  |  |  |
| Mild | 16 (50%) | 7 (29%) | n.s.^†^ |
| Moderate | 10(31%) | 6 (25%) |  |
| Severe | 6 (19%) | 11 (46%) |  |
| HKA (°)^‡^ | -0.43 ± 5.60 | 0.93 ± 8.31 | n.s.^§^ |
| JLCA (°)^‡^ | 0.60 ± 2.56 | 1.64 ± 4.66 | n.s.^∥^ |
| TF subluxation at the lateral cortex (%)^‡^ |  |  |  |
| Knee AP | -3.24 ± 2.45 | -4.19 ± 3.08 | n.s.^§^ |
| Rosenberg view | -1.76 ± 3.07 | -2.42 ± 3.77 | n.s.^∥^ |
| TF subluxation at the center (%)^‡^ |  |  |  |
| Knee AP | -6.14 ± 5.66 | -4.04 ± 6.46 | n.s.^§^ |
| Rosenberg view | -2.42 ± 4.93 | -2.22 ± 7.11 | n.s.^∥^ |
| Hip joint |  |  |  |
| K-L grade of hip^*^ |  |  |  |
| Mild | 26 (81%) | 24 (100%) | n.s.^¶^ |
| Severe | 6 (19%) | 0 (0%) |  |
| HHL (mm)^‡^ | 181.09 ± 7.82 | 186.08 ± 11.44 | n.s.^∥^ |
| FO (°)^‡^ | 43.12 ± 5.13 | 43.10 ± 6.08 | n.s.^§^ |
| NSA (°)^‡^ | 53.92 ± 5.56 | 55.41 ± 5.87 | n.s.^§^ |
| Ankle joint |  |  |  |
| K-L grade of ankle^*^ |  |  |  |
| Mild | 27 (84%) | 24 (100%) | n.s.^¶^ |
| Severe | 5 (16%) | 0 (0%) |  |
| TAA (°)^‡^ | 0.62 ± 2.16 | 0.24 ± 1.50 | n.s.^§^ |
| TTA (°)^‡^ | 1.46 ± 4.26 | 2.43 ± 4.34 | n.s.^∥^ |
| ^*^The values are given as the number of patients.  ^†^Pearson’s chi-square test.  ^‡^The values are given as the mean and the standard deviation.  ^§^Independent t test.  ^∥^Mann-Whitney U test  ^¶^Statistically significant.  ^**^Fisher’s exact test.  *LLD* leg length discrepancy, *HKA* hip-knee-ankle angle, *JLCA* joint line convergence angle, *AP* anteroposterior, *K-L* Kellgren-Lawrence, *HHL* head-head length of pelvis, *FO* femoral offset, *NSA* neck shaft angle of hip, *TAA* tilt angle of ankle, *TTA* tibiotalar angle | | | |
