# Supplementary material for: Decision-making factors and their thresholds for total knee arthroplasty in lateral tibiofemoral osteoarthritis patients: a retrospective cohort study
Source: Knee Surg Relat Res. 2022 Oct 23;34:41. doi: 10.1186/s43019-022-00168-w (PMC9590191; doi:10.1186/s43019-022-00168-w)
Supplement: Supplementary file 2 — Additional file 2. Supplement table 2. [file 43019_2022_168_MOESM2_ESM.docx]

| **Appendix B.** Detecting multicollinearity using variance inflation factor for multivariate logistic regression analysis | | |
| --- | --- | --- |
| Variables | Tolerance | VIF |
| Injection history | 0.711 | 1.406 |
| Ipsilateral side of lower extremity |  |  |
| Pain VAS | 0.675 | 1.481 |
| Ahlbäck grade (mild vs severe) | 0.370 | 2.700 |
| HKA | 0.139 | 7.214 |
| JLCA | 0.271 | 3.692 |
| TF subluxation in the lateral cortex in Rosenberg view | 0.331 | 3.018 |
| TF subluxation in the center in knee AP | 0.479 | 2.086 |
| TTA | 0.759 | 1.318 |
| Contralateral side of lower extremity |  |  |
| Ahlbäck grade (mild vs severe) | 0.675 | 1.481 |
| *VIF* variance inflation factor, *VAS* visual analog scale, *HKA* hip-knee-ankle angle, *JLCA* joint line convergence angle, *AP*; anteroposterior, *TTA* tibiotalar angle | | |
